# Tumor lesions in the supplementary motor area may induce large-scale sensorimotor and cognitive network alterations— A functional and structural connectivity analysis

**DOI:** 10.3389/fneur.2026.1878278

**Published:** 2026-10-21

**Authors:** Chuh-Hyoun Na, Rik Sijben, Oleg Posnansky, Martin Wiesmann, Hans Clusmann, Daniel Delev, Rea Rodriguez-Raecke

**Affiliations:** 1 Department of Neurosurgery, RWTH Aachen University, Aachen, Germany; 2 Center for Integrated Oncology Aachen, Bonn, Cologne, Düsseldorf (CIO ABCD), Aachen, Germany; 3 Brain Imaging Facility, Interdisciplinary Center for Clinical Research (IZKF), RWTH Aachen University, Aachen, Germany; 4 Department of Diagnostic and Interventional Neuroradiology, RWTH Aachen University, Aachen, Germany; 5 Department of Neurosurgery, University of Erlangen, Erlangen, Germany

**Keywords:** brain tumor, connectivity, diffusion tensor imaging (DTI), neuroplasticity, resting-state fMRI (rs-fMRI), SMA-syndrome

## Abstract

**Background:**

Patients with brain tumors involving the SMA often develop postoperatively a mostly transient contralateral akinesia (‘SMA-syndrome'), which may primarily be a cognitive disorder of executive functions. However, the neurophysiological correlates are unclear. Considering the SMA as a gateway between motor and cognitive control functions, we explored structural SMA-connectivity (SC), and functional connectivity (FC) of the sensorimotor (SMN), and of cognitive networks in SMA-tumor patients.

**Methods:**

13 SMA-tumor patients and 13 matched healthy controls underwent diffusion-tensor-imaging (DTI) and resting-state-fMRI preoperatively. Intra- (Frontal Aslant Tract) and interhemispheric SC of the SMA (SMA-SMA, SMA-M1) was compared between groups. FC-networks were established by group Independent Component Analysis (ICA), and subjected to a second-level ROI-to-ROI (region-of-interest) analysis to investigate between-group differences.

**Results:**

DTI revealed microstructural white-matter disintegration and smaller tract volumes of SMA-M1 connectivity (*p* < 0.005), and by trend of the FAT (*p* < 0.06), with differing SMA-M1 fiber-length in patients (*p* < 0.026). SMA-SMA SC was not reduced, but showed greater variance in patients. While FC was significantly decreased within different cognitive networks, FC was increased within the SMN, as well as between the SMA and the contralesional frontoparietal network (*p* < 0.046) in patients.

**Conclusions:**

In this exploratory study, we found sensorimotor- as well as large-scale cognitive network alterations prior to therapeutic intervention, suggesting tumor-associated SC disintegration to be accompanied by in- and decreases of FC within and across different networks. Future studies incorporating neuropsychological-testing are required to characterize pretherapeutic connectivity alterations with regard to their behavioral significance, and to determine whether they may impact the postoperative functional outcome.

## 1 Introduction

SMA tumor patients typically develop postoperatively a 'SMA-syndrome' (1–3), consisting of a contralateral akinesia with or without associated mutism or expressive aphasia, depending on whether the dominant or non-dominant hemisphere is affected. Symptoms commonly regress within a few months, but the time to recovery varies (1), and functional recovery might remain only incomplete (1, 4). Neither the neural correlates of preoperatively preserved motor functions, nor of differing dynamics and degrees of postoperative functional recovery are sufficiently understood, and the individual functional prognosis is difficult to predict. Even though most patients may functionally recover, prolonged neurological deficits can unduly delay adjuvant tumor treatment and can thereby affect the treatment-associated prognosis. A deeper understanding of the neurophysiological prerequisites for early functional recovery is therefore necessary in order to allow maximizing the extent of tumor resection while optimizing the functional outcome, and to improve preoperative counseling of individual patients.

The possibility of complete or nearly complete functional recovery seems to be preserved as long as the primary motor cortex (M1) and the corticospinal tract are not structurally affected. Previous studies suggest a correlation between the time to recovery and the integrity of transcallosal fibers of the sensorimotor network (4–6), and the functional recruitment of the contralateral SMA (7, 8). The postoperative outcome regarding speech has in particular been related to the structural integrity of the left hemispheric Frontal Aslant tract (FAT) (9, 10), connecting the superior frontal gyrus with the ipsilateral inferior frontal gyrus, although FAT preservation was reported to not reliably avoid SMA syndrome postoperatively (11).

Functional connectivity (FC) alterations on the other hand have been described within the sensorimotor network (SMN) in patients with tumor lesions in motor-eloquent or peri-eloquent localizations with decreased interhemispheric FC between the primary motor cortices (M1), even in the absence of motor deficits (12). In a longitudinal study analyzing 6 low grade glioma patients with SMA tumor lesions (8), postoperative motor deficits were associated with reduced ipsilesional as well as interhemispheric resting-state functional MRI (rs-fMRI) connectivity, while functional recovery was accompanied by an increase in FC between the ipsilesional M1 and the contralateral SMA, and with restoration of ipsilesional FC to preoperative levels. However, as no control group was included in this previous study, FC alterations potentially preceding surgical intervention could not be assessed in those patients.

With the SMA being considered as a gateway between motor and cognitive control functions (13–16), the SMA-syndrome has previously been suggested to be primarily a cognitive disorder (17, 18), but the neural correlates and the interrelation between cognitive and motor functions in SMA tumor patients have not been determined. In a first exploratory approach, we investigated functional and structural connectivity in SMA tumor patients compared to healthy controls, hypothesizing that patients would show connectivity alterations in both, the sensorimotor- but as well as cognitive networks, especially with regard to network representations related to cognitive control functions.

## 2 Methods

### 2.1 Participants

SMA tumor patients (mean age: 49 ± 15.4 years, 5 males, 11 right-handed) and 13 age-matched healthy control subjects (mean age: 48 ± 19.7 years, 7 males, 13 right-handed) were included in the study. Handedness was based on the patients' report, but was not formally tested. Between-group differences in age were analyzed using Mann-Whitney–*U*-test, differences regarding sex and handedness were analyzed using Fisher's exact test (significance level *p* < 0.05). Patients were scanned preoperatively, and clinical data was retrospectively evaluated based on patient charts. Clinical data was evaluated preoperatively, as well as early (within the first day postoperatively) and late (median 133 days) postoperatively. For patients' demographics and tumor characteristics please see Table 1. The study was approved by the local ethics committee of the Medical Faculty of the University of the RWTH Aachen (EK142/20) and conducted in accordance with the standards of Good Clinical Practice and the Declaration of Helsinki.

**Table 1:** Patient demographics and tumor characteristics.

Age	Sex	Hand	Site	Histopathology	Tumor grade	CE-tumor	FLAIR	EOR	Last Follow-up	Pre-OP		Post-OP early		Post-OP late	
										M	L	M	L	M	L
56	m	R	L	glioblastoma	V	21.8	81.3	GTR	172	–	+	+	++	–	–
56	f	R	R	glioblastoma	IV	4.0	37.3	GTR	105	–	–	++	–	+	–
77	m	R	R	astrocytoma	III	1.3	37.2	GTR	22	–	–	+++	–	0	0
71	f	L	R	metastasis NSCLC	–	7.4	76.3	GTR	5	+#	–	+	–	0	0
40	f	R	R	metastasis Mamma–CA	–	22.6	90.4	GTR	122	+#	–	+++	–	+	–
42	f	R	R	oligodendroglioma	III	0.3	83.1	GTR	1265	–	–	–	–	–	–
36	f	R	L	astrocytoma	II	4.3	15.5	GTR	84	–	–	+++	++	++	–
81	f	R	L	meningioma	I	20.6	36.3	Simpson G1	612	–	–	–	–	–	–
27	M	R	L	astrocytoma	II	0	47.2	STR	108	–	–	+++	–	+	–
30	m	R	R	oligodendroglioma	III	0.2	85.4	GTR	67	–	–	+++	–	+	–
53	m	R	L	oligodendroglioma	III	0	23.7	SMR	139	–	–	+++	+	–	–
25	f	L	L	astrocytoma	II	0.5	36.8	SMR	133	–	–	+	++	–	–
25	f	R	L	oligodendroglioma	II	0	30.2	SMR	200	–	–	+	+	–	–

Hand, handedness; Site, tumor affected hemisphere; CE-tumor, contrast-enhancing tumor volume given in cm^3^; FLAIR, FLAIR hyperintense tumor lesion given in cm^3^ (corresponding in glioblastoma; metastases and meningioma to perifocal edema, and in grade II and grade III glioma to lower grade tumor lesions); m, male; f, female; R, right; L, left; GTR, gross total resection; Simpson G 1, Simpson Grade I resection: complete macroscopic removal of the meningioma with its dural attachment; STR, subtotal resection; SMR, supramarginal resection, last follow-up given in days; Pre-OP, preoperative clinical status; M, motor symptoms; L, language symptoms; Post-OP, postoperative clinical status, - no symptoms, + mild impairment, ++ moderate impairment, +++ severe impairment; 0, lost to follow-up, #edema, NSCLC, non small cell lung carcinoma; CA, carcinoma.

### 2.2 MRI data acquisition

MRI examination was conducted using a 3T Siemens Prisma MRI scanner equipped with a standard 20-channel head coil. The scanning protocol comprised: a sagittal 3D T1-weighted magnetization-prepared rapid acquisition gradient echo (MPRAGE) sequence [repetition time (TR) = 2,300 ms, echo time (TE) = 2.01 ms, flip angle = 9°, number of slices = 176, voxel size = 1 mm isotropic, and matrix size = 256 × 256, field of view (FoV) = 256 mm], Resting-state-fMRI measurements were acquired with the eyes closed scanning 300 whole brain functional volumes using an echo planar imaging (EPI) sequence [TR = 2,200 ms, TE = 30 ms, flip angle = 90°, number of slices =36, voxel size = 3.1 mm isotropic with a slice gap of 15%, matrix size = 64 x 64, FoV=200 mm]. Due to a scanner software upgrade (syngo MRE11 to syngo MRD13D) which took place during the data acquisition period of the project, diffusion data were acquired using slightly varying parameters. Diffusion data of all healthy controls and 4 patients was acquired using EPI sequences with 64 non-coplanar and non-colinear diffusion directions [*b*-value = 1,000 s/mm^2^, one b0 image with no diffusion weighting for normalization, TR = 6,300 ms, TE = 81 ms, 55 axial slices, voxel size = 2.4 mm isotropic, FoV = 216 mm]. Six patients were measured using a modified sequence [90 diffusion directions, 12 b0 images with no diffusion weighting for normalization, *b*-value = 1,000 s/mm^2^, TR = 6,900 ms, TE = 53 ms, 74 axial slices, voxel size = 2.0 mm isotropic, FoV = 256 mm]. One participant was measured with most parameters identical to the latter with a varying TR of 7,400 ms. Differences in acquisition parameters were accounted for during data processing. Importantly, scanner performance before and after the upgrade was systematically evaluated using the ACR phantom according to standardized quality assurance procedures. The phantom measurements demonstrated stable or improved scanner performance after the upgrade, including preserved geometric accuracy, slice positioning, spatial resolution, image uniformity, and reduced ghosting artifacts, all within ACR tolerance limits.

### 2.3 Structural MRI

Based on contrast enhanced T1-weighted and FLAIR weighted structural MRI, Tumor lesion volumes were segmented using brainlab analysis software (Anatomical Mapping/SmartBrush, v4.1, Brainlab AG, Munich, Germany, http://www.brainlab.com), distinguishing contrast enhancing tumor +/- necrosis (CE-tumor) and FLAIR hyperintense tumor lesions, which were classified as a) edema in glioblastoma/metastases/meningioma, and b) as non-enhancing tumor in grade II and III gliomas.

### 2.4 Preprocessing

Diffusion acquisitions were corrected for susceptibility-induced distortions, and for eddy currents and motion artifacts using the advanced normalization tools (ANTS) (19, 20). Images' deformations were restricted to the phase-encoding axis following the standard procedure for EPI distortion correction. The resulting deformation field map was applied to the diffusion images to shift the voxels in the areas affected by distortion. Diffeomorphic registration was performed using the nonlinear SyN algorithm of ANTS. The same preprocessing pipeline was applied to all diffusion datasets, including correction for susceptibility-induced distortions, eddy currents, and motion. Although this standardized preprocessing reduces variability attributable to differences in image-processing procedures, it cannot fully eliminate systematic differences originating from the acquisition protocols themselves. Diffusion metrics (FA, MD, AD, RD) were subsequently derived from tensor fitting. T1 images were segmented into WM, gray matter and CSF using DSI Studio (https://dsi-studio.labsolver.org/). The tissue segmentation, as well as the AAL parcellation (21) were transformed into diffusion space using nonrigid diffeomorphic image registration of T1 image and diffusion image. To support group level analysis of ipsi- and contralesional connections, tumor lateralization was standardized by left-right flipping images of patients with right hemispheric lesions (*n* = 7). For consistency, a matching number of images were flipped in the control data set.

Functional images were preprocessed using the default pipeline of the CONN functional connectivity toolbox for SPM12, version 20b (22, 23) based on MATLAB 2020^1^. This preprocessing pipeline included realignment of functional images to the mean functional volume, unwarping, slice-time correction, and outlier detection using artifact detection tools (ART). EPI-volumes were co-registered to T1 images, and structural and functional images were normalized to MNI152-space and resliced to a voxel size of 2 mm isotropic. As part of the normalization gray-matter, white-matter and cerebrospinal fluid were segmented. Correct segmentation and normalization of the images, especially those obtained from the patient group, was visually verified. Functional images were smoothed with an isotropic kernel of 8 mm full width at half-maximum. After basic pre-processing, functional images were further denoised by regressing out confounds based on signals recorded from white matter and CSF, realignment parameters, and detected outliers. Data was also whitened to reduce autocorrelations, and finally a 0.008 Hz (1/125s) high-pass filter was applied. To minimize the risk of artificial anti-correlations, component-based correction without global signal regression was performed: maximum and mean head motion was compared between patients and controls using Mann–Whitney *U*–tests. Patients and controls did not show any significant differences regarding maximum (*U* = 86.0, *p* =.960) or mean (*U* = 87.0, *p* = 0.920) head motion. Exclusion of outliers did not change this outcome.

### 2.5 DTI analyses

Deterministic diffusion tractography was performed in native diffusion space using anatomical constraints of the transformed tissue segmentation map. The b-table was checked by an automatic quality control routine to ensure its accuracy (24). The diffusion tensor was calculated which allowed to apply a deterministic fiber tracking algorithm (25). For nonlinear registration of the AAL2 based parcellation to the native space we used DSI Studio. The tract-seed was assigned to the pars opercularis of the inferior frontal gyrus, and the tract-end was assigned to the SMA on either hemisphere. Analyses were repeated flipping the seed and end. Fibers were obtained with a deterministic fiber tracking algorithm as implemented by Yeh et al. (25) with a step size randomly selected between 0.5 and 1.5 voxels and a maximum angle between subsequent steps randomly selected between 15 and 90°. Tracking seed points were set to the boundary between gray matter and WM using 50,000 seed points per voxel. Tracking was terminated if the FA value was below a default value (0.112432) when the WM boundary was reached. All stream-lines that did not terminate in gray matter or where the final length was less than 30 mm or longer than 300 mm were discarded. As interpolation is required to produce a visually continuous fiber pathway due to the typically coarse spatial resolution, interpolation errors might be introduced. Also, when fiber tracts with different orientations coexist in a voxel, the model based on a single tensor becomes inadequate. For such cases fibers were visually inspected and erroneous singular fibers manually cut off. While interrater-variability was not accounted for, we sought to minimize false-positive tracts through rigorous, blinded refinement using strict anatomical exclusion criteria.

The total number of streamlines connecting both regions (“number of fibers”), tract volume, tract length as well as means of fractional anisotropy (FA), mean diffusivity (MD), axial diffusivity (AD), and radial diffusivity (RD) were determined for each subject in either hemisphere.

Statistical analyses of diffusion-parameters were performed with SPSS 28^2^. Firstly, mean values were calculated for each of the parameters, and averaged over tracking directions. Mean values for fiber length, FA, MD, AD, and RD were weighted by the number of tracts generated per tracking direction. The number of fibers and tract volume were both normalized by division of the mean size of the seeding and ending regions used for fiber tracking. Extreme outliers, defined by a 3 inter-quartile-range criterion, were excluded. Any remaining deviations from normality were relatively minor with neither skewness nor kurtosis exceeding critical values. Parameters of fibers connecting bilateral SMA (SMA-SMA) were analyzed using independent samples *t*-tests, comparing patients to healthy controls. Parameters of fibers connecting SMA to ipsilateral IFG (FAT) or contralateral M1 (SMA-M1) were analyzed using SPSS' mixed procedure, comparing patients to healthy controls, and treating lateralization (ipsi- or contralesional) as a repeated measure. Additionally, an interaction term of the two factors was included in both models. All statistical comparisons were tested two-sided with a significance level of *p* < 0.05. Considering the only small sample size and the explorative character of the present studies, uncorrected significant interactions are reported here and were investigated further using *post-hoc* pairwise comparisons, applying Bonferroni-corrections to control the family-wise error rate, but results can therefore only be regarded as trends, requiring validation in future studies including larger sample sizes. Results of the *t*-tests are reported using mean values and standard deviations. Results of the mixed models are reported using marginal means and corresponding standard errors.

### 2.6 Resting-state fMRI functional connectivity analyses

Unbiased network templates were generated using a group independent component analysis (ICA) analysis as implemented in the CONN functional connectivity toolbox. Forty group level independent components were obtained by running FastICA with a G1 contrast function (26) followed by a GICA3 back-projection (27, 28) with a subject level dimensionality reduction of 64. The 40 components were then inspected and labeled based on which resting state network they best represented. Labeling was guided by a spatial correlation to template based on the Harvard-Oxford and AAL atlases. As ICA provided mostly network components rather than comprehensive network representations, spatial correlation coefficients or the dice coefficient for indicating the spatial overlap were not computed. Spatial maps of 14 components corresponding to the central executive network, dorsal attention network, salience network, SMN, and DMN were used for further analyses and subjected to a second-level ROI-to-ROI connectivity analysis for investigating between-group differences, using a hierarchical clustering algorithm based on differences in connectivity statistics and spatial criteria as implemented in CONN. The significance threshold was set at *p* < 0.05 (FDR corrected).

## 3 Results

### 3.1 Demographics and structural MRI

Controls were not matched in education (as this information was not available in patients based on the retrospective analysis), but were matched in age, handedness and sex. Patients and controls did not differ in age (mean age controls: 49 ± 15.4 years, mean age patients: 47.6 ± 19.7 years, Mann–Whitney-*U* = 74.5, *p* = 0.64). Regarding sex, five male and eight female subjects were in the patient group, while seven males and six females were in the control group. Using Fisher's exact test showed no significant difference between groups (*p* = 0.695). With regard to handedness, controls were all right-handed, while two of the patients were left-handed, but both groups did not differ significantly in handedness (Fisher's exact test, *p* = 0.480) (for details please see Table 1). Right-hemispheres were flipped onto the left hemisphere when the tumor was in the right-hemisphere, so that connectivity of lesioned and non-lesioned hemispheres could be analyzed across patients. As six patients had right-hemispheric, and seven patients had left-hemispheric tumor lesion, the number of right and left hemispheres was balanced within the patient group. For between-group comparisons, a corresponding number of right hemispheres were accordingly flipped onto the left hemisphere in controls, so that the number of right-and left hemispheres was balanced across groups.

While contrast-enhancing tumor volumes (CE-tumor, please see as well Table 1) did not differ between hemispheres (mean left: 6.74 ± 10 cm^3^, mean right: 5.98 ± 8.6 cm^3^, Mann–Whitney-*U* = 16.0, *p* = 0.518), FLAIR-hyperintense lesion volumes were larger in right-hemispheric tumor lesions (mean left: 38.7 ± 21.3 cm^3^, mean right: 68.3 ± 24.5 cm^3^, Mann–Whitney-*U* = 5.0, *p* = 0.022). However, when taking into account the total lesion volume (sum of contrast-enhancing tumor and FLAIR hyperintense lesion volume, please see Table 1), right and left-hemispheric lesion volumes did not differ (mean left: 45.5 ± 28.5 cm^3^, mean right: 74.3 ± 28.9 cm^3^, Mann–Whitney-*U* = 11.0, *p* = 0.101) between hemispheres.

### 3.2 Clinical findings

Only two of the patients showed preoperatively minor motor deficits, and only one patient minor speech impairment (reduced verbal fluency) prior to surgery. Early postoperatively, the majority of patients (85%) developed a SMA syndrome with contralateral akinesia and additional speech deficits in 5 (out of 8 left-hemispheric) tumor patients (38%). At follow-up (median 133 days), motor deficits improved in the majority (81%, *n* = 9) and speech deficits in all patients, albeit with incomplete motor recovery observed in 55% (*n* = 6) of patients at the time of follow-up (s. Table 1).

### 3.3 Intra- and interhemispheric structural connectivity of the SMA

#### 3.3.1 Frontal aslant tract (FAT)

For FA, we observed a trend for lateralization (F(1, 20.993) = 4.253, *p* = 0.052) with higher FA values in the contralesional hemisphere across groups. Analysis of MD (F(1, 21.177) = 4.739, *p* = 0.041) and AD F(1, 21.164) = 6.155, *p* = 0.022) revealed a significant interaction, with increased values for the ipsilesional as compared to the contralesional FAT only in patients (MD: mean difference = 0.065, SE = 0.021, t(21.683) = 3.1, *p* =.012; AD: mean difference = 0.061, SE = 0.019, t(21.785) = 3.2, *p* = 0.01), suggesting structural disintegration within the ipsilesional hemisphere of patients. A similar trend was found for RD, with the interaction falling just short of significance (please see Figure 1 and Supplementary Tables S1, S2).

**Figure 1:**
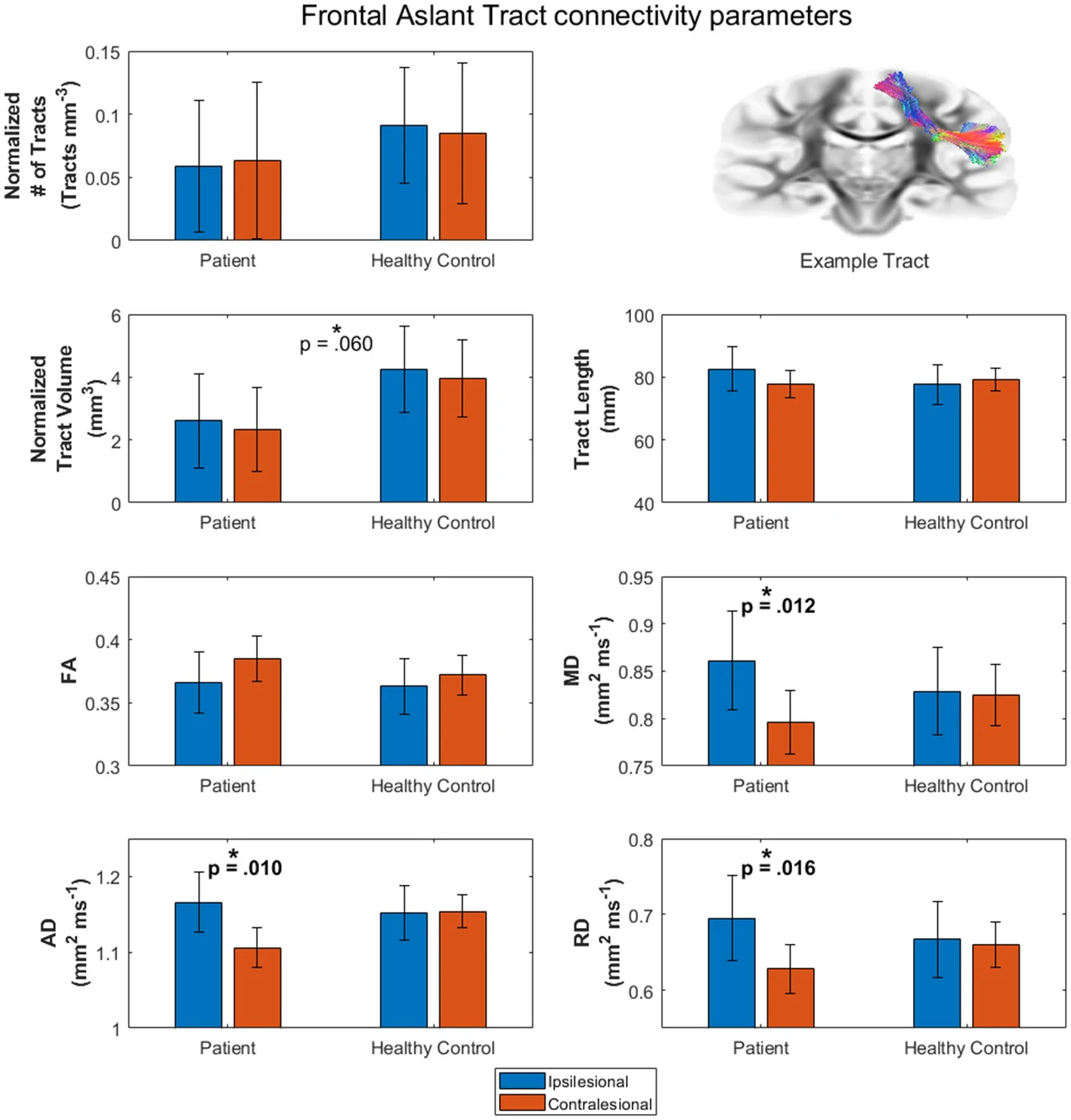
Mean tract characteristics and diffusion parameters of the FAT. Error bars depict two SD (standard deviations). All statistical comparisons were tested two-sided with a significance level of *p* < 0.05. Correction for multiple comparisons was not applied in view of the small sample size. Significant interactions were investigated further using *post-hoc* pairwise comparisons, applying Bonferroni-corrections to control the family-wise error rate, *p* < 0.05. See Supplementary Tables S1, S2 for detailed statistics. *marks significant differences or trends.

#### 3.3.2 Interhemispheric SMA-M1 connectivity

We analyzed heterologue transcallosal connectivity between the ipsilesional SMA and the contralesional M1 (ipsilesional SMA-M1), as well as between the contralesional SMA and the ipsilesional M1 (contralesional SMA-M1).

Normalized tract volumes were overall significantly reduced in patients (patients *M* = 1.686, SE = 0.546; controls *M* = 4.045, SE = 0.526: F(1, 21.605) = 9.682, *p* = 0.005), which was independent of hemisphere. Analysis of fiber lengths revealed a significant interaction (F(1, 19.425) = 6.197, *p* = 0.022), showing longer contralesional SMA-M1 fibers as compared to ipsilesional SMA-M1 fibers in patients (mean difference = −24.308, SE = 8.849, t(18.139) = 2.7, *p* = 0.026), but not in controls (mean difference = 7.411, SE = 9.166, t(20.415) = 0.8, *p* = 0.856).

Significant group differences were found for AD (*p* < 0.019) and by trend for MD (*p* < 0.066), suggesting higher mean values in patients independent of hemisphere. Although a similar tendency was observed with regard to RD, differences were not significant here (see Figure 2 and Supplementary Tables S3, S4). Regarding FA, neither significant differences nor interactions were found

**Figure 2:**
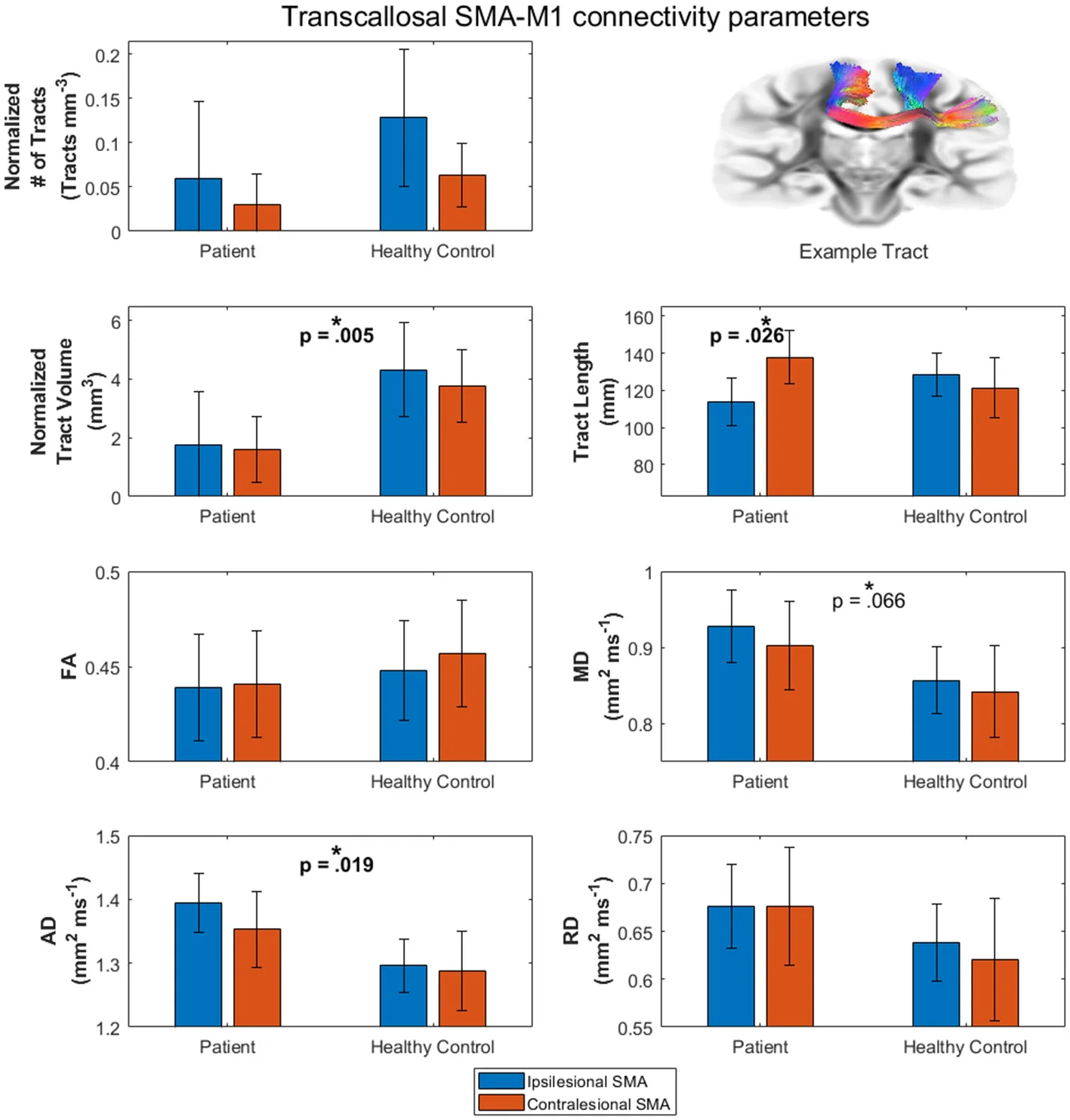
Mean tract characteristics and diffusivity parameters of SMA-M1 connectivity. Error bars depict 2 SD. All statistical comparisons were tested two-sided with a significance level of *p* < 0.05. Correction for multiple comparisons was not applied in view of the small sample size. Significant interactions were investigated further using *post-hoc* pairwise comparisons, applying Bonferroni-corrections to control the family-wise error rate, *p* < 0.05. See Supplementary Tables S3, S4 for detailed statistics. *marks significant differences or trends.

#### 3.3.3 Interhemispheric SMA-SMA connectivity

As opposed to SMA-M1 and FAT, SC of homolog transcallosal SMA was not reduced in patients as compared to controls (please see Figure 3, for detailed statistics please see Supplementary Tables S5, S6). Mean normalized number of tracts seemed to be even rather increased in some of the patients (*M* = 0.81, SD = 0.47) as compared to healthy controls (*M* = 0.56, SD = 0.34), but without reaching statistical significance due to the higher variance in patients. We therefore further explored the patient data, comparing *post hoc* (independent samples *t*-test, equal variances not assumed, two-sided) those patients with *no motor deficit* (*n* = 5) to those with residual *motor deficits* (*n* = 6) at the time of follow-up. While mean number of fibers seemed to be relatively higher in the no deficit group (*M* = 1.024, SD = 0.37 vs. *M* = 0.6270, SD = 0.486), differences were not statistically significant (*p* < 0.155), but with a trend toward higher SMA-SMA tract volumes (*p* < 0.058) in the *no motor deficit* as compared to the *deficit group* (*M* = 16.59, SD = 6.9 vs. *M* = 8.5, SD = 3.3).

**Figure 3:**
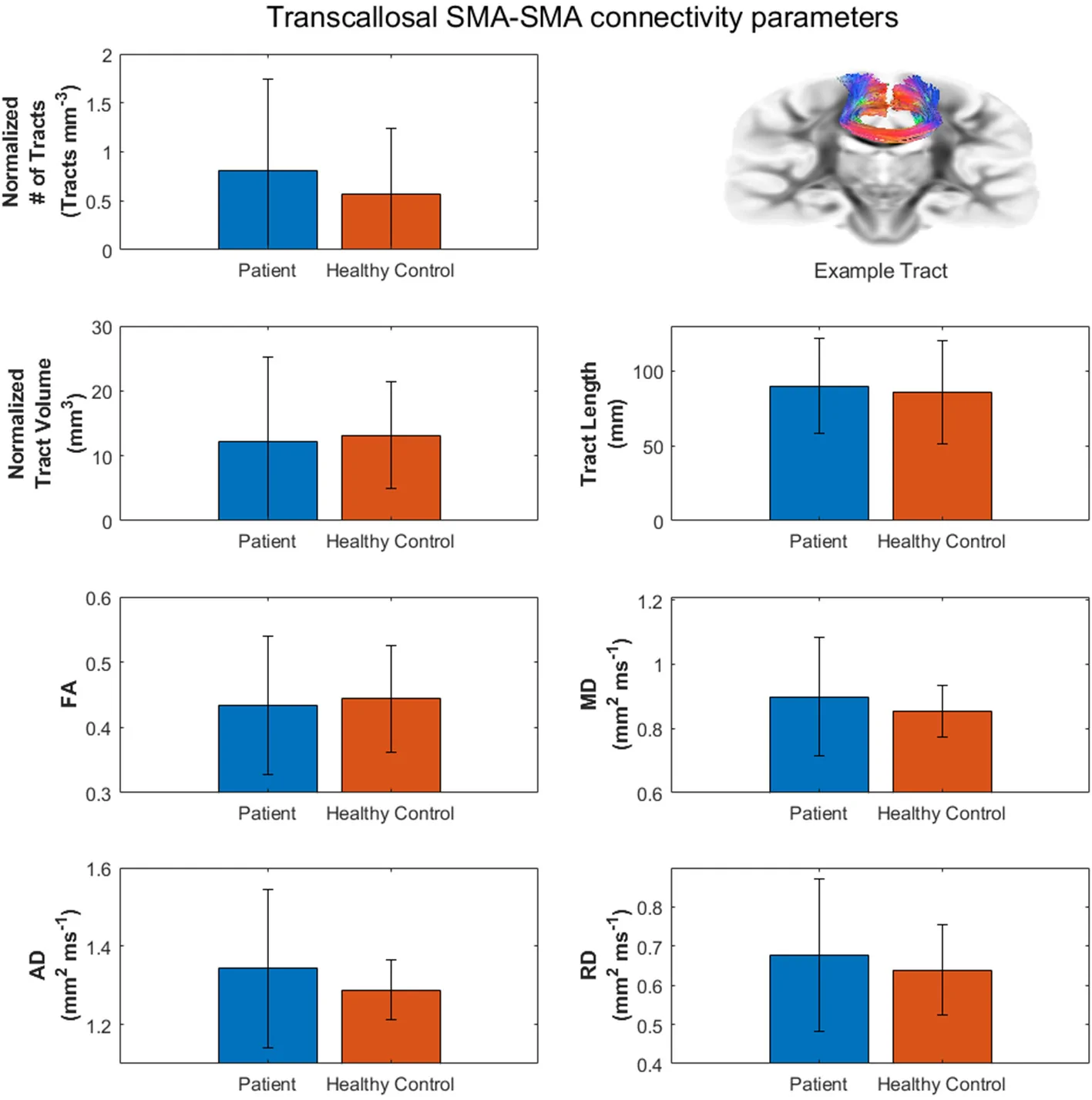
Mean tract characteristics and diffusivity parameters of transcallosal SMA-SMA connectivity. Error bars depict two SD (standard deviations). All statistical comparisons were tested two-sided with a significance level of *p* < 0.05. Correction for multiple comparisons was not applied in view of the small sample size. Significant interactions were investigated further using *post-hoc* pairwise comparisons, applying Bonferroni-corrections to control the family-wise error rate, *p* < 0.05. See Supplementary Tables S4, S5 for detailed statistics.

However, when correcting for multiple comparisons (applying the Benjamini Hochberg procedure for FDR correction), some of the interactions did not remain significant (please find detailed values in the table of the Supplementary material), which might be related to the limited statistical power of the only small sample size. The present study can therefore not exclude the risk of false-positive findings, and considering the significant simple effects of the interactions only at an uncorrected level, the present findings require validation in future studies including larger sample sizes.

### 3.4 Functional connectivity

Independent component analysis (ICA) segregated 40 different functional network components, out of which 14 independent components (IC) of interest relating to either the sensorimotor or cognitive networks (anterior, posterior and lateral DMN, right and left FPN, anterior and frontostriatal components of the salience network, dorsal and ventral attention, and frontomedian network components) were chosen for further analyses (Figure 4A) (please see spatial files of the selected 14 components as Supplementary material).

**Figure 4:**
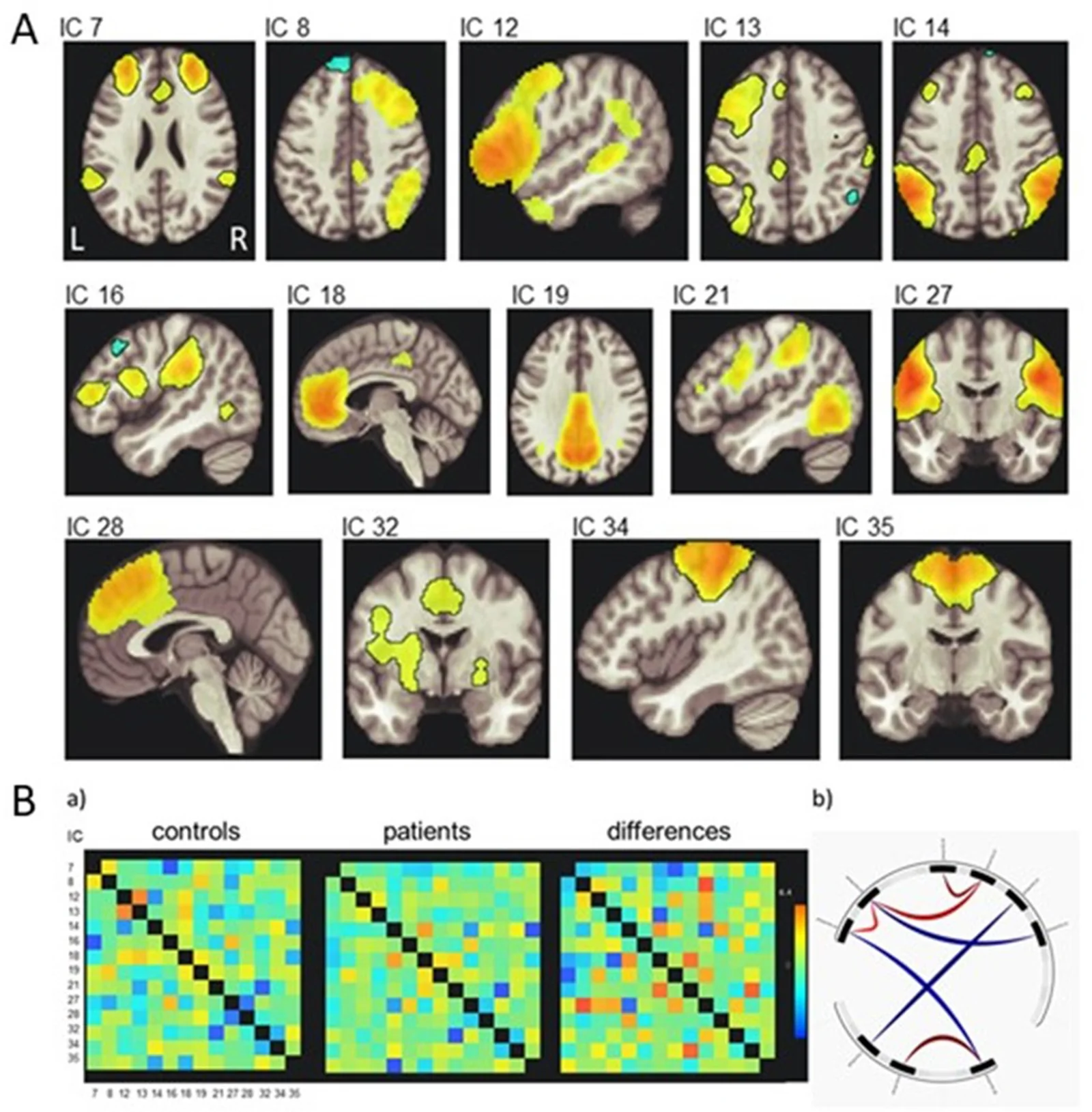
**(A):** Independent components (IC) used for analyses. IC7/32: anterior/frontostriatal salience network components, IC8/13: right/left frontoparietal network, IC12/16/21: ventral and dorsal attention network components, IC14/18/19: lateral/anterior/dorsal DMN, IC28: frontomedian component, IC32: frontostriatal network, IC 27/34/35: lateral/dorsal/medial sensorimotor network components. (**B): (**a) Mean connectivity matrices of ROI-to-ROI functional connectivity analyses of different ICs, each of controls and patients and between-groups differences. (b) Significant differences in interconnectivity of ICs across groups extracted in a connectogram, with *red* indicating increased and *blue* decreased functional connectivity in patients compared to controls (significance threshold set at *p* < 0.05, FDR corrected).

Subjecting these 14 ICs to an ROI-to-ROI internetwork-connectivity analyses revealed three connectivity clusters in which patients and controls significantly differed (*p* < 0.05, FDR corrected) (Figure 4B).

Using a hierarchical clustering algorithm based on temporal (differences in mean time series) and spatial criteria (differences in local distribution), three different clusters of significantly altered internetwork-connectivity were identified across groups:

*Cluster 1* [F(2, 23) = 4.47, FDR corrected *p* < 0.046] showed decreased coupling of the lateral DMN and the frontomedian network, as well as decreased connectivity of dorsal attention and salience network components in patients (Figure 5).

**Figure 5:**
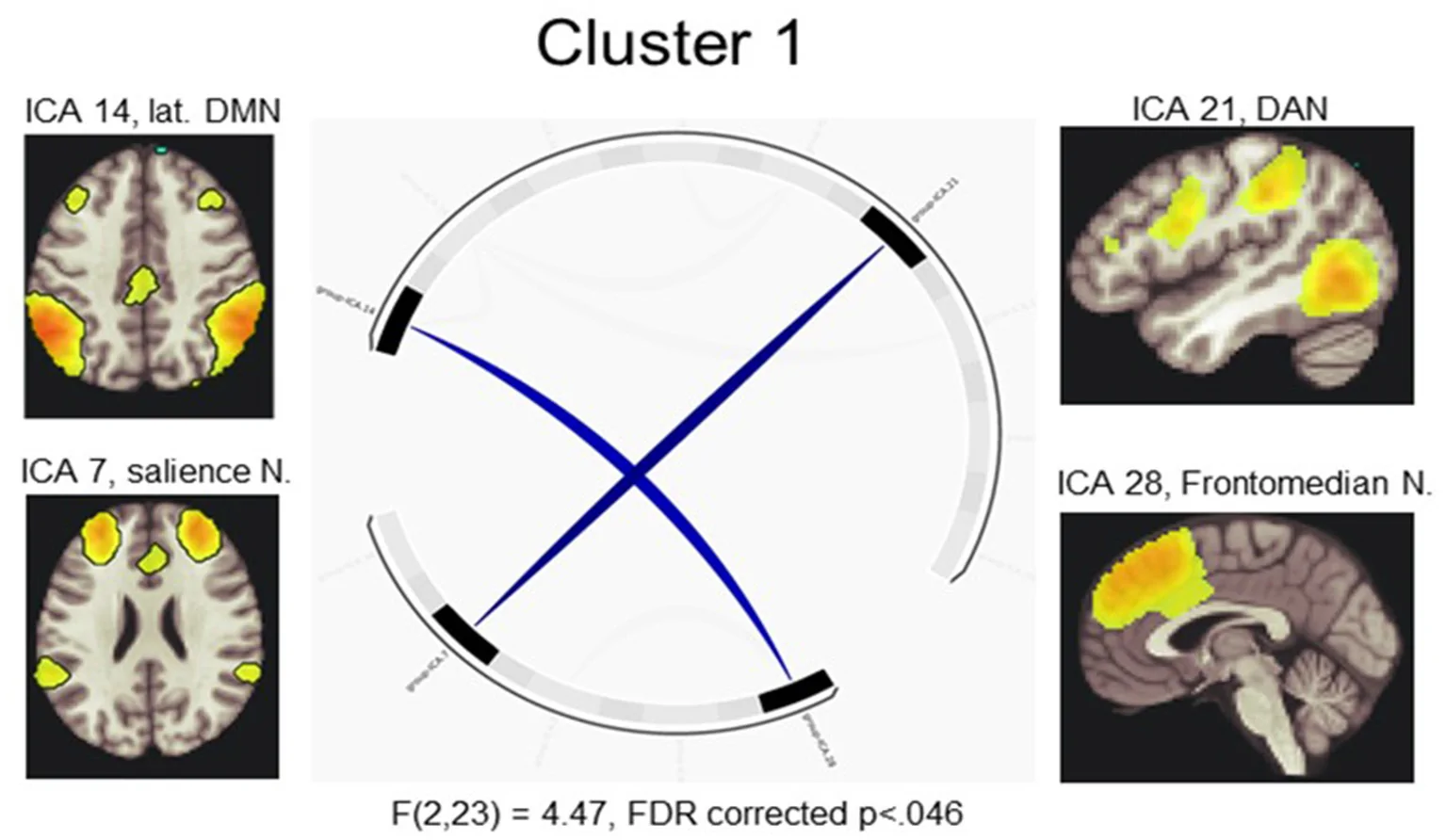
Connectogram of cluster 1 showing significantly (significance threshold set at *p* < 0.05, FDR corrected) decreased functional connectivity (blue) across different network components in patients compared to controls.

*Cluster 2* [F(2, 23) = 3.85, FDR corrected *p* < 0.046 showed decreased connectivity of the ipsilesional FPN to the anterior DMN, while increased connectivity was found for the anterior and lateral DMN, the anterior DMN and the lateral SMN, as well as between medial and lateral SMN components (Figure 6).

**Figure 6:**
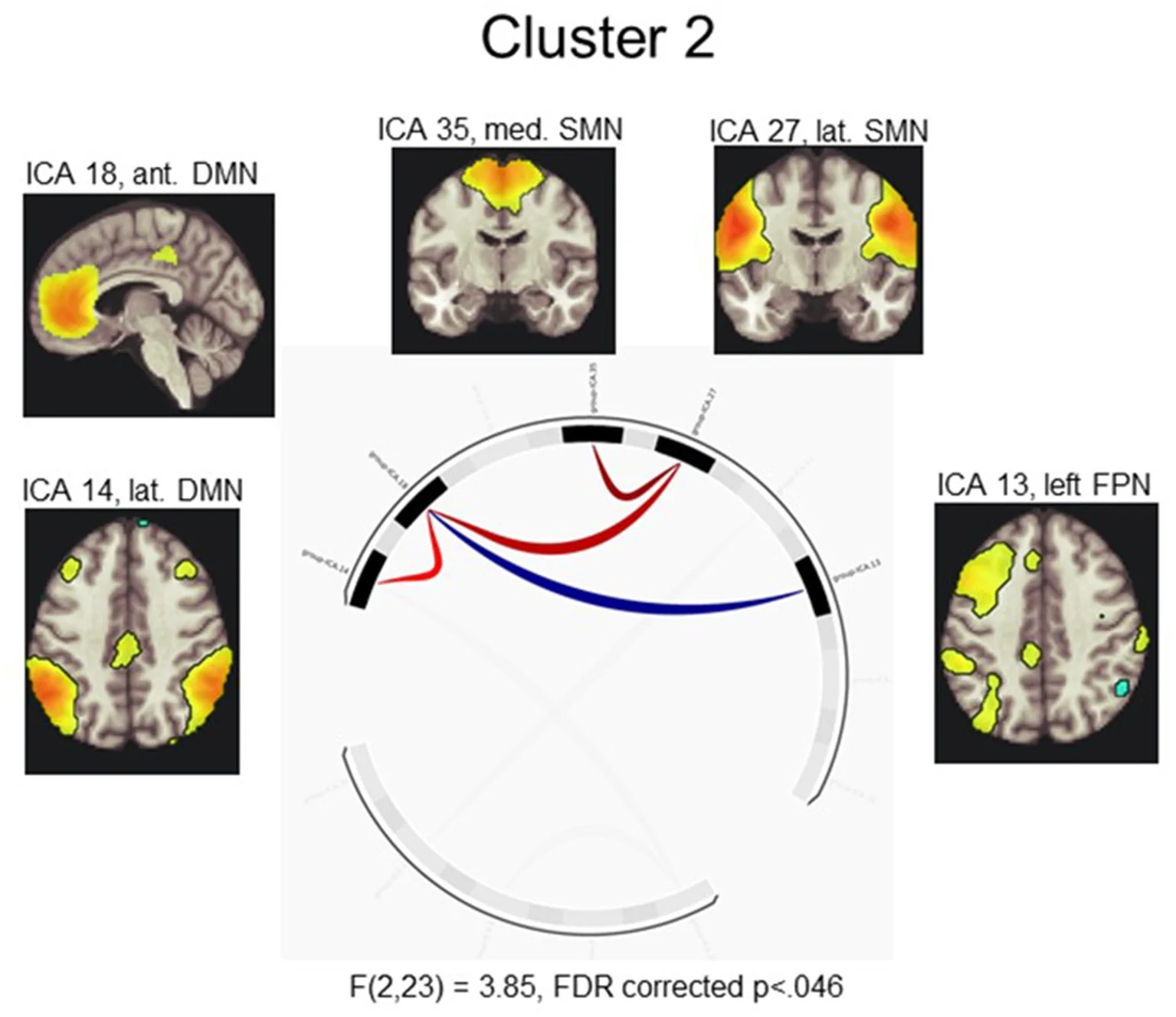
Connectogram of cluster 2 showing significantly decreased (blue) and increased (red) functional connectivity (significance threshold set at *p* < 0.05, FDR corrected) across different network components in patients compared to controls.

*Cluster 3* [F(2, 23) = 3.54, FDR corrected *p* < 0.046] showed increased coupling of the contralesional FPN and the frontomedian network including the SMA (Figure 7).

**Figure 7:**
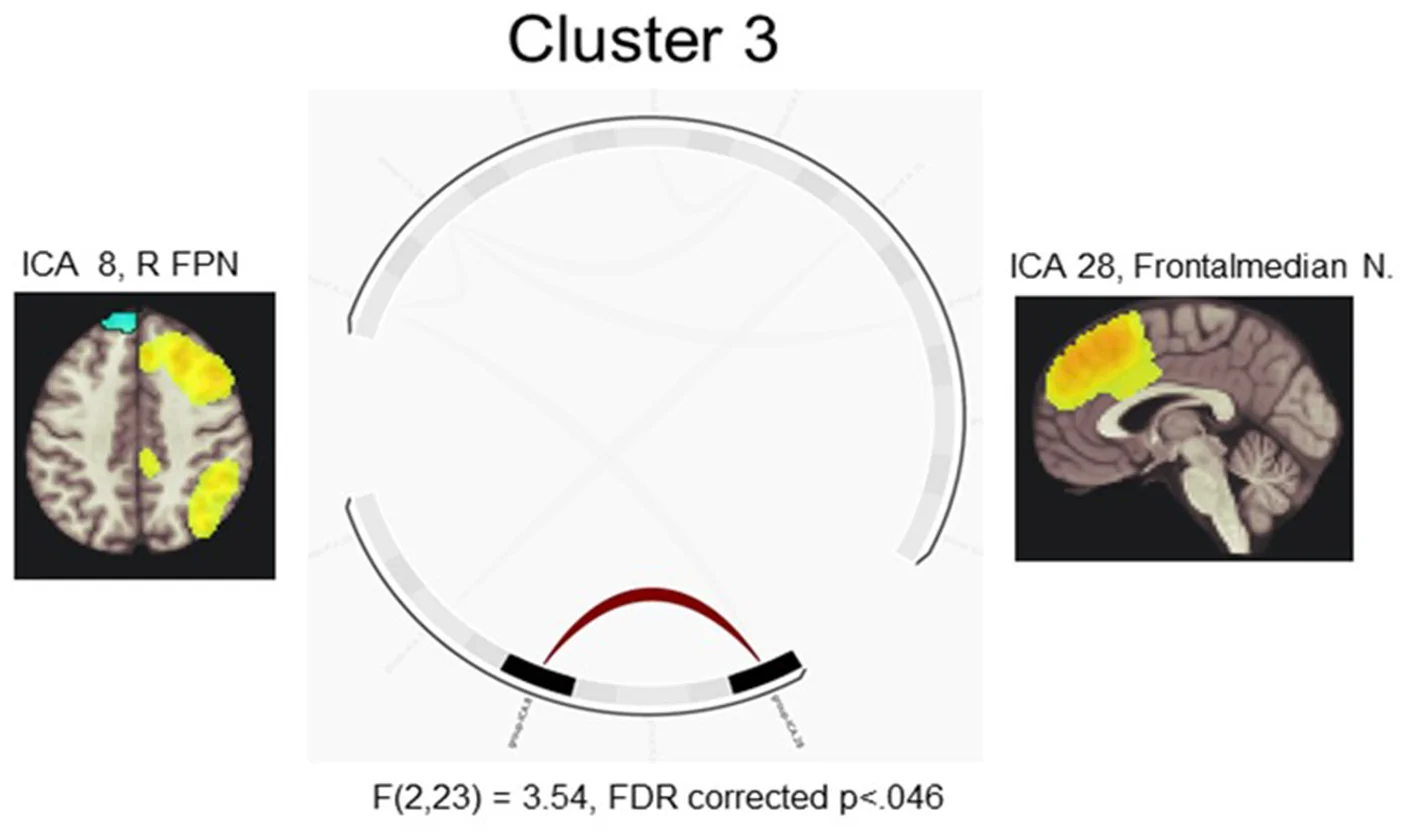
Connectogram of cluster 3 showing significantly increased (red) (significance threshold set at *p* < 0.05, FDR corrected) functional connectivity across different network components in patients compared to controls.

## 4 Discussion

We found functional and structural connectivity alterations in SMA tumor patients prior to therapeutic intervention. Patients showed not only signs of microstructural fiber tract disintegration and FC decreases, but also greater variance in homolog structural transcallosal SMA connectivity, accompanied by increased functional coupling within the SMN, within the DMN, between the SMA and the DMN, as well as between the SMA and the contralesional FPN. These connectivity alterations may not only indicate tumor associated alterations of the sensorimotor network, but might also relate to altered cognitive functions, which we could however not evidence in the present study, as no neuropsychological data had been obtained. Even though only exploratory in character, our findings may suggest, that functional connectivity alterations within and across different domains may already precede therapeutic intervention in SMA tumor patients, which however requires validation in larger studies.

### 4.1 Motor control and the SMA

Considering the crucial role of the SMA in the initiation of voluntary movements with evidence of vast reciprocal connections of the SMA to cortical- and subcortical structures, as well as to the cerebellum, it has been proposed to be a gateway “to stored subroutines” of previously acquired motor programs (29). As the SMA is involved in different neurocircuits (30), SMA-tumor associated connectivity alterations are likely to affect not only the sensorimotor network, but as well those cognitive networks subserving preparation and cognitive control of motor functions. In particular, the pre-SMA and the SMA proper as subregions of the SMA have different anatomical connectivity and functional roles in motor execution, motor initiation, language, and cognitive control. Although mapping tumor lesions relative to SMA proper vs. pre-SMA could improve anatomical interpretation, a distinction between the pre-SMA and the SMA (proper which is usually based on probabilistic maps of healthy individuals or individual fMRI data) was not attempted in our tumor patients, as -besides the macroscopically apparent tumor lesions- regular cytoarchitecture and thus distinct individual pre-SMA/SMA-proper projections and boundaries are likely to have also been altered by the tumor lesions. However, our findings seem to comply with the notion, that the SMA subserves multiple networks across different domains, as we found increased functional coupling not only within and between the DMN and the SMN, but as well between the SMA and the contralesional FPN. However, as no neuropsychological testing had been performed, we can neither define, whether functional connectivity alterations were indeed related to altered cognitive control of motor functions, nor can we determine, whether FC increases were functionally beneficial or rather detrimental based on the present data.

### 4.2 Cognitive dysfunction in SMA tumor patients

Higher cognitive functions other than language (31) have only scarcely been investigated in SMA tumor patients, although neuroanatomical and neuroimaging studies suggest an involvement of the SMA especially in executive processes. Cañas et al. (32) suggested cognitive dysfunction in SMA tumor patients to exceed pure language deficits, and Sjöberg (18) reported significantly impaired cognitive control functions in SMA tumor patients. However, the neural correlates of cognitive dysfunction or their potential impact on motor functions have not yet been determined. Neuropsychological testing should therefore be systematically performed in patients with tumors involving the SMA, as a better understanding of the cognitive impairment might open new therapeutic targets for the behavioral re- or even prehabilitation of the SMA syndrome, e.g., by establishing cognitive treatment strategies.

### 4.3 Intra- and interhemispheric structural SMA connectivity

#### 4.3.1 Intrahemispheric SMA connectivity

The Frontal Aslant Tract has particularly been linked to verbal fluency and initiation of speech (33–35). Previous studies suggest lesioning of the left FAT in SMA tumor patients to be related to postoperative speech impairment (10, 36), while preservation of the FAT has been related to a more favorable postoperative functional outcome (9). Our data shows microstructural disintegration of the ipsilesional FAT to exceed the macroscopically apparent tumor localization already pretherapeutically.

#### 4.3.2 Interhemispheric structural connectivity of the SMA

Oda et al. (5) previously reported significant differences in the time to postoperative functional recovery in SMA syndrome, depending on the number of transcallosal fibers connecting the contralesional SMA with the ipsilesional M1. Baker et al. (4) furthermore suggested transcallosal SMA to SMA and premotor cortex connections as the so called 'crossed FAT' to potentially subserve postoperative clinical recovery in SMA tumor patients. We found signs of microstructural white-matter disintegration also for interhemispheric SMA-M1 connectivity, with tract volumes tending to be reduced in patients as compared to healthy controls. Tract length was found to significantly differ interhemispherically only in patients, showing increased tract length between the contralesional SMA to the ipsilesional M1, as compared to the ipsilesional SMA to the contralesional M1, which might relate to tumor-lesion associated displacement and distension of surrounding fiber structures in the ipsilesional hemisphere.

However, unlike for FAT and SMA-M1 connectivity, we found no significant structural disintegration for SMA-SMA connectivity in patients. The number of fibers seemed to be even rather increased in some of the patients, which, however, did not reach statistical significance due to the greater variance in patients. Comparing those patients with, to those without residual postoperative motor deficits showed a trend toward larger SMA-SMA tract volumes in those patients with, as compared to those without complete clinical recovery of motor symptoms, although formal inferences are precluded considering the only small number of subjects. While our present findings can only be regarded as explorative hypothesis-generating, preoperatively increased or at least better preserved structural transcallosal homologuous SMA connectivity might be critical for functional recovery, by providing a better anatomical basis for bihemispheric and/or compensatory contralateral neural processing (5).

### 4.4 Connectomic alterations preceding therapeutic intervention

While functional recovery after stroke or trauma has been related to neuroplasticity following acute brain injury, the chronic disease course in brain tumor patients is likely to be accompanied by neural connectivity alterations of both, disintegrative as well as compensatory nature, even prior to disease manifestation. Functional reorganization or even neuroplasticity accompanying the chronic disease course in tumor patients may allow to maintain functional abilities, as long as the tumor-induced structural disintegration does not outweigh compensatory capacities, which are more likely to be longer preserved in slower growing, and non- or less infiltrative tumor lesions. However, the degree of tumor-induced structural and functional connectivity changes is likely to substantially differ depending on the growth velocity, and on the infiltrative/non-infiltrative growth pattern of a tumor. We would expect infiltrative and faster growing tumor lesions to cause earlier structural disruption with less functional reorganization, while non- or less infiltrative and slower growing tumor lesions are more likely to be associated with less structural disintegration and with a higher possibility of functional reorganization and -in some patients perhaps even- plasticity. The degree and nature of connectomic alterations preceding therapeutic intervention therefore might also influence the early postoperative functional outcome, as patients with a higher degree of functional reorganization and less structural desintegration at the time of surgical intervention are more likely to have a better functional outcome after tumor resection than those without. Characterizing and understanding the functional significance of connectomic alterations prior to surgical intervention could therefore be highly relevant for a better prognostication of the individual functional outcome, and to advance individual treatment strategies.

### 4.5 Limitations and perspectives

A major limitation of the present studies is the only small sample size restricting statistical power, as well as the lack of neuropsychological test metrics, which did not allow to actually assess the behavioral correlates of the connectivity alterations found. Furthermore, tumor patients were heterogenous in tumor types, as lesion location was the primary inclusion criterion here. Given the exploratory study character with an only limited sample size and due to the retrospective study nature, tumor- and clinical characteristics as well as antiepileptic medication were not specifically accounted for in the present study. We therefore cannot exclude that these factors may have influenced the analysis results. Considering the heterogeinity of our tumor cohort, the only borderline findings and the high variance observed regarding homolog SMA connectivity in our study might be related to such heterogeinity in tumor growth behavior, and in potentially resulting differences in tumor-induced connectomic alterations. Larger patient samples with homogenous tumor entities are therefore required to investigate assumed differences in connectomic alterations between patients with fast and slow growing tumor lesions, which could not be addressed in the present study.

Based on the observation, that patients did not have substantial motor or speech impairment preoperatively despite considerable tumor volumes and signs of structural disintegration, we considered the observed increase in functional connectivity as potentially compensatory. However, based on the present findings, we cannot determine, whether functional connectivity increases were indeed behaviourally beneficial. Pathological hyperactivity, corresponding to tumor-neural coupling, functional disinhibition, or tumor associated alterations in neurovascular coupling cannot be excluded, and prospective studies including behavioral data including larger samples are required to investigate the behavioral relevance of such functional connectivity alterations.

A further limitation of the DTI data is, that deterministic tractography algorithms are inherently vulnerable to false negatives in regions with crossing fibers, significant tumor distortion, or severe edema. This limitation seems particularly relevant for the reconstruction of the FAT and transcallosal SMA-M1 pathways, where fiber crossing can lead to premature termination of tracking or aberrant pathway steering. While advanced probabilistic or multi-fiber reconstruction algorithms and employing multi-shell diffusion data might offer higher sensitivity in these regions, they are more prone to false-positives. Moreover, multi-shell data acquisitions usually require longer acquisition times, which we tried to avoid in patients. Furthermore, the rather conservative estimation of fiber connections based on the deterministic tractography algorithms seemed preferable to prevent overestimation of fiber counts. However, inter-rater reliability was not assessed, and the potential underestimation of tract volume due to tumor-induced signal degradation remains a limitation of the DTI analysis. An additional limitation concerns differences in the diffusion acquisition protocol among some of the subjects resulting from a scanner software upgrade during the study period. We therefore cannot completely exclude a contribution of protocol-related effects to FA, MD, AD, RD, or tractography-derived measures. Analysis was limited by the small number of patients and should therefore be interpreted cautiously, as acquisition protocol and patient group were not fully balanced, preventing complete statistical separation of these effects. Although non-uniformly, structural connectivity of the homologous transcallosal SMA-SMA pathway was not reduced as opposed to SMA-M1 or FAT connectivity in patients, this finding cannot necessarily be interpreted as evidence against potential acquisition-related confounding.

However, despite these limitations and although only explorative in character, our study is one of the rare studies characterizing both, functional as well as structural connectivity alterations in SMA tumor patients. Our data suggest, that pretherapeutic connectomic alterations should be further investigated in tumor patients. Prospective longitudinal studies should evaluate, whether characterizing pretherapeutic functional and structural connectivity alterations may aid to improve prognostication of the postoperative functional outcome, which –if validated in future studies- could aid in improving treatment planning and individual patient counseling.

## 5 Conclusion

SMA-tumor patients showed structural as well as large-scale functional connectivity alterations already prior to therapeutic interventions. These connectivity alterations may not only indicate tumor-associated structural and functional disintegration, but might also relate to patterns compatible with possible intra- and internetwork reorganization within and across domains, which we could however not evidence based on the present, only exploratory study. Results of the present study require validation in larger samples, multicenter cohorts, and longitudinal follow-up studies. It should be investigated in future studies, whether pretherapeutic connectomic alterations might be associated with altered cognitive control of motor functions in SMA tumor patients, and whether these might impact the postoperative functional outcome.

## Data Availability

The datasets presented in this article are not publicly available because of ethical and privacy restrictions. Requests to access converted and anonymized data may be provided upon request.

## Footnotes

1 MATLAB. Published online 2020.

2 SPSS. Published online 2021.

## References

[1] Palmisciano P Haider AS Balasubramanian K Dadario NB Robertson FC Silverstein JW . Supplementary motor area syndrome after brain tumor surgery: a systematic review. World Neurosurg. (2022) 165:160–71. doi: 10.1016/j.wneu.2022.06.080 35752423

[2] Pinson H Van Lerbeirghe J Vanhauwaert D Van Damme O Hallaert G Kalala JP. The supplementary motor area syndrome: a neurosurgical review. Neurosurg Rev. (2022) 45:81–90. doi: 10.1007/s10143-021-01566-6 33993354

[3] Zentner J Hufnagel A Pechstein U Wolf HK Schramm J. Functional results after resective procedures involving the supplementary motor area. J Neurosurg. (1996) 85:542–9. doi: 10.3171/jns.1996.85.4.0542 8814153

[4] Baker CM Burks JD Briggs RG Smitherman AD Glenn CA Conner AK . The crossed frontal aslant tract: a possible pathway involved in the recovery of supplementary motor area syndrome. Brain Behav. (2018) 8:e00926. doi: 10.1002/brb3.926 29541539 PMC5840439

[5] Oda K Yamaguchi F Enomoto H Higuchi T Morita A. Prediction of recovery from supplementary motor area syndrome after brain tumor surgery: preoperative diffusion tensor tractography analysis and postoperative neurological clinical course. Neurosurg Focus. (2018) 44:E3. doi: 10.3171/2017.12.FOCUS17564 29852764

[6] Tuncer MS Fekonja LS Ott S Pfnür A Karbe AG Engelhardt M . Role of interhemispheric connectivity in recovery from postoperative supplementary motor area syndrome in glioma patients. J Neurosurg. (2022) 139:324–33. doi: 10.3171/2022.10.JNS221303 36461815

[7] Acioly MA Cunha AM Parise M Rodrigues E Tovar-Moll F. Recruitment of contralateral supplementary motor area in functional recovery following medial frontal lobe surgery: an fMRI case study. J Neurol Surg A Cent Eur Neurosurg. (2015) 76:508–12. doi: 10.1055/s-0035-1558408 26291886

[8] Vassal M Charroud C Deverdun J Le Bars E Molino F Bonnetblanc F . Recovery of functional connectivity of the sensorimotor network after surgery for diffuse low-grade gliomas involving the supplementary motor area. J Neurosurg. (2017) 126:1181–90. doi: 10.3171/2016.4.JNS152484 27315027

[9] Briggs RG Allan PG Poologaindran A Dadario NB Young IM Ahsan SA . The frontal aslant tract and supplementary motor area syndrome: moving towards a connectomic initiation axis. Cancers. (2021) 13:1116. doi: 10.3390/cancers13051116 33807749 PMC7961364

[10] Kinoshita M de Champfleur NM Deverdun J Moritz-Gasser S Herbet G Duffau H. Role of fronto-striatal tract and frontal aslant tract in movement and speech: an axonal mapping study. Brain Struct Funct. (2015) 220:3399–412. doi: 10.1007/s00429-014-0863-0 25086832

[11] Young JS Gogos AJ Aabedi AA Morshed RA Pereira MP Lashof-Regas S . Resection of supplementary motor area gliomas: revisiting supplementary motor syndrome and the role of the frontal aslant tract. J Neurosurg. (2021) 136:1278–84. doi: 10.3171/2021.4.JNS21187 34598138

[12] Niu C Zhang M Min Z Rana N Zhang Q Liu X . Motor network plasticity and low-frequency oscillations abnormalities in patients with brain gliomas: a functional MRI study. PLoS ONE. (2014) 9:e96850. doi: 10.1371/journal.pone.0096850 24806463 PMC4013133

[13] Lehéricy S Ducros M Krainik A Francois C Van de Moortele PF Ugurbil K . 3-D diffusion tensor axonal tracking shows distinct SMA and pre-SMA projections to the human striatum. Cerebral cortex. (2004) 14:1302–9. doi: 10.1093/cercor/bhh091 15166103

[14] Liu J Morel A Wannier T Rouiller EM. Origins of callosal projections to the supplementary motor area (SMA): a direct comparison between pre-SMA and SMA-proper in macaque monkeys. J Comp Neurol. (2002) 443:71–85. doi: 10.1002/cne.10087 11793348

[15] Roth JK Johnson MK Tokoglu F Murphy I Constable RT. Modulating intrinsic connectivity: adjacent subregions within supplementary motor cortex, dorsolateral prefrontal cortex, and parietal cortex connect to separate functional networks during task and also connect during rest. PLoS ONE. (2014) 9:e90672. doi: 10.1371/journal.pone.0090672 24637793 PMC3956459

[16] Ruan J Bludau S Palomero-Gallagher N Caspers S Mohlberg H Eickhoff SB . Cytoarchitecture, probability maps, and functions of the human supplementary and pre-supplementary motor areas. Brain Struct Funct. (2018) 223:4169–86. doi: 10.1007/s00429-018-1738-6 30187192 PMC6267244

[17] Sjöberg RL. Free will and neurosurgical resections of the supplementary motor area: a critical review. Acta Neurochir. (2021) 163:1229–37. doi: 10.1007/s00701-021-04748-9 33566193 PMC8053652

[18] Sjöberg RL Stålnacke M Andersson M Eriksson J. The supplementary motor area syndrome and cognitive control. Neuropsychologia. (2019) 129:141–5. doi: 10.1016/j.neuropsychologia.2019.03.013 30930302

[19] Avants BB Tustison N Song G. Advanced normalization tools (ANTS). Insight J. (2009) 2:1–35. doi: 10.54294/uvnhin

[20] Avants BB Tustison NJ Song G Cook PA Klein A Gee JC . Reproducible evaluation of ANTs similarity metric performance in brain image registration. Neuroimage. (2011) 54:2033–44. doi: 10.1016/j.neuroimage.2010.09.025 20851191 PMC3065962

[21] Rolls ET Huang CC Lin CP Feng J Joliot M. Automated anatomical labelling atlas 3. Neuroimage. (2020) 206:116189. doi: 10.1016/j.neuroimage.2019.116189 31521825

[22] Eickhoff SB Stephan KE Mohlberg H Grefkes C Fink GR. Amunts K, et al. A new SPM toolbox for combining probabilistic cytoarchitectonic maps and functional imaging data. Neuroimage. (2005) 25:1325–35. doi: 10.1016/j.neuroimage.2004.12.034 15850749

[23] Nieto-Castanon A. Handbook of Functional Connectivity Magnetic Resonance Imaging Methods *in CONN*. Boston, MA: Hilbert Press. (2020).

[24] Schilling KG Blaber J Huo Y Newton A Hansen C Nath V . Synthesized b0 for diffusion distortion correction (Synb0-DisCo). Magn Reson Imaging. (2019) 64:62–70. doi: 10.1016/j.mri.2019.05.008 31075422 PMC6834894

[25] Yeh FC Verstynen TD Wang Y Fernández-Miranda JC Tseng WYI. Deterministic diffusion fiber tracking improved by quantitative anisotropy. PLoS ONE. (2013) 8:e80713. doi: 10.1371/journal.pone.0080713 24348913 PMC3858183

[26] Hyvarinen A. Fast and robust fixed-point algorithms for independent component analysis. IEEE Trans Neural Netw. (1999) 10:626–34. doi: 10.1109/72.761722 18252563

[27] Calhoun VD Adali T Pearlson GD Pekar JJ. A method for making group inferences from functional MRI data using independent component analysis. Hum Brain Mapp. (2001) 14:140–51. doi: 10.1002/hbm.1048 11559959 PMC6871952

[28] Erhardt EB Rachakonda S Bedrick EJ Allen EA Adali T Calhoun VD. Comparison ofmulti-subject ICAmethods for analysis of fMRI data (Erratumin: Hum Brain Mapp. 2024 Aug 15;45(12):e70006. doi: 10.1002/hbm.70006.) *Hum Brain Mapp*. (2011) 32:2075–95. doi: 10.1002/hbm.21170 PMC3117074 21162045

[29] Eccles JC. The initiation of voluntary movements by the supplementary motor area. Arch Psychiatr Nervenkr. (1982) 231:423–41. doi: 10.1007/BF00342722 6812546

[30] Eccles JC. Some circuit operations in the mammalian brain. Cells Tissues Organs. (1982) 113:325–39. doi: 10.1159/000145567 6295045

[31] Tymowski M Kaspera W Metta-Pieszka J Zarudzki Ł Ładziński P. Neuropsychological assessment of patients undergoing surgery due to low-grade glioma involving the supplementary motor area. Clin Neurol Neurosurg. (2018) 175:1–8. doi: 10.1016/j.clineuro.2018.09.036 30292977

[32] Cañas A Juncadella M Lau R Gabarrós A Hernández M. Working memory deficits after lesions involving the supplementary motor area. Front Psychol. (2018) 9:765. doi: 10.3389/fpsyg.2018.00765 29875717 PMC5974158

[33] Catani M. Dell'Acqua F, Vergani F, Malik F, Hodge H, Roy P, et al. Short frontal lobe connections of the human brain. Cortex. (2012) 48:273–91. doi: 10.1016/j.cortex.2011.12.001 22209688

[34] Catani M Mesulam MM Jakobsen E Malik F Martersteck M Wieneke C . A novel frontal pathway underlies verbal fluency in primary progressive aphasia. Brain. (2013) 136:2619–28. doi: 10.1093/brain/awt163 23820597 PMC3722349

[35] La Corte E Eldahaby D Greco E Aquino D Bertolini G Levi V . The frontal aslant tract: a systematic review for neurosurgical applications. Front Neurol. (2021) 12:641586. doi: 10.3389/fneur.2021.641586 33732210 PMC7959833

[36] Ille S Engel L Kelm A Meyer B Krieg SM. Language-eloquent white matter pathway tractography and the course of language function in glioma patients. Front Oncol. (2018) 8:572. doi: 10.3389/fonc.2018.00572 30574455 PMC6291459

