# Supplementary material for: Tumor lesions in the supplementary motor area may induce large-scale sensorimotor and cognitive network alterations— A functional and structural connectivity analysis
Source: Front Neurol. 2026 Oct 21;17:1878278. doi: 10.3389/fneur.2026.1878278 (PMC13732028; doi:10.3389/fneur.2026.1878278)
Supplement: Supplementary file 1 [file Data_Sheet_1.pdf]

Uncorrected FAT

DTI measure	Patient main effect	Side/Analysis main effect	Patient × Side interaction
# tracts	0,341	0,969	0,846
Tract volume	0,06	0,574	0,999
Tract length	0,601	0,46	0,16
FA	0,567	0,052	0,49
MD	0,958	**0,023**	**0,041**
AD	0,37	**0,03**	**0,022**
RD	0,93	**0,024**	0,06

Uncorrected SMA-M1

DTI measure	Patient main effect	Side/Analysis main effect	Patient × Side interaction
# tracts	0,133	0,145	0,573
Tract volume	**0,005**	0,611	0,791
Tract length	0,921	0,2	**0,022**
FA	0,478	0,452	0,676
MD	0,066	0,21	0,728
AD	**0,019**	0,199	0,376
RD	0,173	0,578	0,601

Uncorrected SMA-SMA

Measure	Patient main effect
# tracts	0,159
Tract volume	0,675
Tract length	0,592
FA	0,615
MD	0,17
AD	0,118
RD	0,268

HBFDR FAT

DTI measure	Patient main effect	Side/Analysis main effect	Patient × Side interaction
# tracts	0,841	0,969	0,987
Tract volume	0,42	0,67	0,999
Tract length	0,841	0,644	0,28
FA	0,841	0,091	0,686
MD	0,958	0,07	0,14
AD	0,841	0,07	0,14
RD	0,958	0,07	0,14

HBFDR SMA-M1

DTI measure	Patient main effect	Side/Analysis main effect	Patient × Side interaction
# tracts	0,233	0,368	0,791
Tract volume	**0,035**	0,611	0,791
Tract length	0,921	0,368	0,154
FA	0,558	0,611	0,791
MD	0,154	0,368	0,791
AD	0,067	0,368	0,791
RD	0,242	0,611	0,791

HBFDR SMA-SMA

Measure	Patient main effect
# tracts	0,397
Tract volume	0,675
Tract length	0,675
FA	0,675
MD	0,397
AD	0,397
RD	0,469
