# Supplementary material for: Tumor lesions in the supplementary motor area may induce large-scale sensorimotor and cognitive network alterations— A functional and structural connectivity analysis
Source: Front Neurol. 2026 Oct 21;17:1878278. doi: 10.3389/fneur.2026.1878278 (PMC13732028; doi:10.3389/fneur.2026.1878278)
Supplement: Supplementary file 3 [file Table_1.docx]

**Supplement**

**Table 1**

| **Frontal Aslant Tract (FAT) connectivity statistics**  **Linear mixed model with within-subject correlations** | | | | | |
| --- | --- | --- | --- | --- | --- |
|  | | **df1** | **df2** | **F** | **p** |
| Normalized number of tracts | |  |  |  |  |
|  | Group | 1 | 23.676 | 0.946 | .341 |
|  | Lateralization | 1 | 23.377 | 0.002 | .969 |
|  | Group * Lateralization | 1 | 23.377 | 0.038 | .846 |
| Normalized tract volume | |  |  |  |  |
|  | Group | 1 | 22.598 | 3.923 | .060 |
|  | Lateralization | 1 | 20.816 | 0.327 | .574 |
|  | Group * Lateralization | 1 | 20.816 | 0.000 | .999 |
| Tract length | |  |  |  |  |
|  | Group | 1 | 22.478 | 0.282 | .601 |
|  | Lateralization | 1 | 21.734 | 0.566 | .460 |
|  | Group * Lateralization | 1 | 21.734 | 2.113 | .160 |
| Fractional anisotropy | |  |  |  |  |
|  | Group | 1 | 22.876 | 0.338 | .567 |
|  | Lateralization | 1 | 20.993 | 4.253 | .052 |
|  | Group * Lateralization | 1 | 20.993 | 0.493 | .490 |
| Mean diffusivity | |  |  |  |  |
|  | Group | 1 | 14.314 | 0.003 | .958 |
|  | Lateralization | 1 | 21.177 | 6.057 | .023 |
|  | Group * Lateralization | 1 | 21.177 | 4.739 | .041 |
| Axial diffusivity | |  |  |  |  |
|  | Group | 1 | 20.827 | 0.839 | .370 |
|  | Lateralization | 1 | 21.164 | 5.429 | .030 |
|  | Group * Lateralization | 1 | 21.164 | 6.155 | .022 |
| Radial diffusivity | |  |  |  |  |
|  | Group | 1 | 17.196 | 0.008 | .930 |
|  | Lateralization | 1 | 21.282 | 5.950 | .024 |
|  | Group * Lateralization | 1 | 21.282 | 3.954 | .060 |

Table S1. Statistical overview of fiber parameters connecting SMA to ipsilateral IFG. Df1 = degrees of freedom numerator, df2 = degrees of freedom denominator.

**Table 2**

| **Frontal Aslant Tract (FAT) connectivity – Pairwise comparisons** | | | | | |
| --- | --- | --- | --- | --- | --- |
|  | **Group** | **Lateralization** | **Mean** | **SE** | **p** |
| Normalized number of tracts | Patient | Ipsilesional | 0.059 | 0.026 | >1.00 |
|  |  | Contralesional | 0.063 | 0.031 |  |
|  | Control | Ipsilesional | 0.091 | 0.023 | >1.00 |
|  |  | Contralesional | 0.085 | 0.028 |  |
| Normalized tract volume | Patient | Ipsilesional | 2.608 | 0.752 | .124 |
|  |  | Contralesional | 2.324 | 0.664 |  |
|  | Control | Ipsilesional | 4.245 | 0.689 | .083 |
|  |  | Contralesional | 3.962 | 0.613 |  |
| Tract length | Patient | Ipsilesional | 82.580 | 3.538 | .322 |
|  |  | Contralesional | 77.608 | 2.162 |  |
|  | Control | Ipsilesional | 77.619 | 3.088 | >1.00 |
|  |  | Contralesional | 79.199 | 1.887 |  |
| Fractional Anisotropy | Patient | Ipsilesional | 0.366 | 0.012 | .170 |
|  |  | Contralesional | 0.385 | 0.009 |  |
|  | Control | Ipsilesional | 0.363 | 0.011 | .608 |
|  |  | Contralesional | 0.372 | 0.008 |  |
| Mean diffusivity | Patient | Ipsilesional | 0.861 | 0.026 | .012* |
|  |  | Contralesional | 0.796 | 0.017 |  |
|  | Control | Ipsilesional | 0.829 | 0.023 | >1.00* |
|  |  | Contralesional | 0.825 | 0.016 |  |
| Axial Diffusivity | Patient | Ipsilesional | 1.166 | 0.020 | .010* |
|  |  | Contralesional | 1.106 | 0.013 |  |
|  | Control | Ipsilesional | 1.152 | 0.018 | >1.00* |
|  |  | Contralesional | 1.154 | 0.011 |  |
| Radial Diffusivity | Patient | Ipsilesional | 0.695 | 0.028 | 0.016 |
|  |  | Contralesional | 0.628 | 0.016 |  |
|  | Control | Ipsilesional | 0.667 | 0.025 | >1.00 |
|  |  | Contralesional | 0.660 | 0.015 |  |
| *P-values correspond to the simple effects of lateralization and are Bonferroni corrected. Values exceeding 1 are reported as >1.00. * Indicates a significant interaction term.* | | | | | |

Table S2. Pairwise comparisons of fiber parameters of the frontal aslant tract. SE = standard error.

**Table 3**

| **Transcallosal SMA-M1 connectivity statistics**  **Linear mixed model with within-subject correlations** | | | | | |
| --- | --- | --- | --- | --- | --- |
|  | | **df1** | **df2** | **F** | **p** |
| Normalized number of tracts | |  |  |  |  |
|  | Group | 1 | 20.200 | 2.449 | .133 |
|  | Lateralization | 1 | 20.096 | 2.305 | .145 |
|  | Group * Lateralization | 1 | 20.096 | 0.328 | .573 |
| Normalized tract volume | |  |  |  |  |
|  | Group | 1 | 21.605 | 9.682 | .005 |
|  | Lateralization | 1 | 21.093 | 0.267 | .611 |
|  | Group * Lateralization | 1 | 21.093 | 0.072 | .791 |
| Tract length | |  |  |  |  |
|  | Group | 1 | 19.811 | 0.010 | .921 |
|  | Lateralization | 1 | 19.425 | 1.759 | .200 |
|  | Group * Lateralization | 1 | 19.425 | 6.197 | .022 |
| Fractional anisotropy | |  |  |  |  |
|  | Group | 1 | 23.996 | 0.520 | .478 |
|  | Lateralization | 1 | 19.403 | 0.589 | .452 |
|  | Group * Lateralization | 1 | 19.403 | 0.181 | .676 |
| Mean diffusivity | |  |  |  |  |
|  | Group | 1 | 24.263 | 3.703 | .066 |
|  | Lateralization | 1 | 20.001 | 1.680 | .210 |
|  | Group * Lateralization | 1 | 20.001 | 0.125 | .728 |
| Axial diffusivity | |  |  |  |  |
|  | Group | 1 | 24.217 | 6.325 | .019 |
|  | Lateralization | 1 | 20.525 | 1.758 | .199 |
|  | Group * Lateralization | 1 | 20.525 | 0.817 | .376 |
| Radial diffusivity | |  |  |  |  |
|  | Group | 1 | 23.647 | 1.972 | .173 |
|  | Lateralization | 1 | 17.817 | 0.320 | .578 |
|  | Group * Lateralization | 1 | 17.817 | 0.283 | .601 |

Table S5. Statistical overview of transcallosal fiber parameters connecting SMA to contralateral M1. Df1 = degrees of freedom numerator, df2 = degrees of freedom denominator.

**Table 4**

| **Transcallosal SMA-M1 connectivity – Pairwise comparisons** | | | | | |
| --- | --- | --- | --- | --- | --- |
|  | **Group** | **Lateralization** | **Mean** | **SE** | **p** |
| Normalized number of tracts | Patient | Ipsilesional | 0.060 | 0.043 | >1.00 |
|  |  | Contralesional | 0.030 | 0.017 |  |
|  | Control | Ipsilesional | 0.128 | 0.039 | .280 |
|  |  | Contralesional | 0.063 | 0.018 |  |
| Normalized tract volume | Patient | Ipsilesional | 1.774 | 0.892 | >1.00 |
|  |  | Contralesional | 1.598 | 0.563 |  |
|  | Control | Ipsilesional | 4.323 | 0.807 | >1.00 |
|  |  | Contralesional | 3.767 | 0.622 |  |
| Tract length | Patient | Ipsilesional | 113.611 | 6.426 | .026* |
|  |  | Contralesional | 137.919 | 7.311 |  |
|  | Control | Ipsilesional | 128.713 | 5.824 | .856* |
|  |  | Contralesional | 121.303 | 8.046 |  |
| Fractional Anisotropy | Patient | Ipsilesional | 0.439 | 0.014 | >1.00 |
|  |  | Contralesional | 0.441 | 0.014 |  |
|  | Control | Ipsilesional | 0.448 | 0.013 | .840 |
|  |  | Contralesional | 0.457 | 0.014 |  |
| Mean diffusivity | Patient | Ipsilesional | 0.928 | 0.024 | .480 |
|  |  | Contralesional | 0.902 | 0.029 |  |
|  | Control | Ipsilesional | 0.857 | 0.022 | >1.00 |
|  |  | Contralesional | 0.842 | 0.030 |  |
| Axial Diffusivity | Patient | Ipsilesional | 1.395 | 0.023 | .234 |
|  |  | Contralesional | 1.353 | 0.030 |  |
|  | Control | Ipsilesional | 1.296 | 0.021 | >1.00 |
|  |  | Contralesional | 1.288 | 0.031 |  |
| Radial Diffusivity | Patient | Ipsilesional | 0.676 | 0.022 | >1.00 |
|  |  | Contralesional | 0.676 | 0.031 |  |
|  | Control | Ipsilesional | 0.638 | 0.020 | .920 |
|  |  | Contralesional | 0.620 | 0.032 |  |
| *P-values correspond to the simple effects of lateralization and are Bonferroni corrected. Values exceeding 1 are reported as >1.00. * Indicates a significant interaction term.* | | | | | |

Table S6. Pairwise comparisons of transcallosal fiber parameters connecting SMA to contralateral M1. SE = standard error.

| **Table 5**  **Transcallosal SMA-SMA connectivity statistics**  **Independent student’s t-test (Patient > Healthy Control)** | | | |
| --- | --- | --- | --- |
|  | **t** | **df** | **p** |
| Normalized number of tracts | 1.459 | 21 | .159 |
| Normalized tract volume | -0.426 | 21 | .675 |
| Tract length | 0.545 | 21 | .592 |
| Fractional anisotropy | -0.510 | 21 | .615 |
| Mean diffusivity | 1.449 | 13.4 | .170^a^ |
| Axial diffusivity | 1.738 | 12.5 | .118^a^ |
| Radial diffusivity | 1.137 | 21 | .268 |
|  | | | |
|  | | | |
| ^a^ Levene’s test is significant, Welsch’s t-test reported instead to  compensate inequal variance. | | | |

Table S3. Independent t-test results, comparing parameters of transcallosal fibers connecting bilateral SMA in patients and healthy controls. Df = degrees of freedom.

**Table 6**

| **Transcallosal SMA-SMA connectivity descriptives** | | | | | |
| --- | --- | --- | --- | --- | --- |
|  | **Group** | **N** | **Mean** | **SD** | **SE** |
| Normalized number of tracts | P | 11 | 0.810 | 0.467 | 0.141 |
|  | HC | 12 | 0.563 | 0.341 | 0.099 |
| Normalized tract volume | P | 11 | 12.200 | 6.516 | 1.965 |
|  | HC | 12 | 13.160 | 4.132 | 1.193 |
| Tract length | P | 11 | 89.850 | 15.880 | 4.788 |
|  | HC | 12 | 86.070 | 17.260 | 4.983 |
| Fractional anisotropy | P | 11 | 0.434 | 0.053 | 0.016 |
|  | HC | 12 | 0.444 | 0.041 | 0.012 |
| Mean diffusivity | P | 11 | 0.899 | 0.092 | 0.028 |
|  | HC | 12 | 0.855 | 0.040 | 0.011 |
| Axial diffusivity | P | 11 | 1.343 | 0.101 | 0.030 |
|  | HC | 12 | 1.288 | 0.038 | 0.011 |
| Radial diffusivity | P | 11 | 0.677 | 0.098 | 0.030 |
|  | HC | 12 | 0.639 | 0.058 | 0.017 |

Table S4. Descriptive statistics of transcallosal fiber parameters connecting bilateral SMA.P = Patient, HC = Healthy control, SD = Standard deviation, SE = standard error.
